# Breast density as indicator for the use of mammography or MRI to screen women with familial risk for breast cancer (FaMRIsc): a multicentre randomized controlled trial

**DOI:** 10.1186/1471-2407-12-440

**Published:** 2012-10-02

**Authors:** Sepideh Saadatmand, Emiel J T Rutgers, RobAEM Tollenaar, Hermien M Zonderland, MargreetGEM Ausems, KristienBMI Keymeulen, Margreet S Schlooz-Vries, Linetta B Koppert, Eveline A M Heijnsdijk, Caroline Seynaeve, Cees Verhoef, Jan C Oosterwijk, Inge-Marie Obdeijn, Harry J de Koning, Madeleine M A Tilanus-Linthorst

**Affiliations:** 1 Department of Surgery, Erasmus University Medical Centre, Rotterdam, Netherlands; 2 Department of Surgery, The Netherlands Cancer Institute, Antoni van Leeuwenhoek Hospital, Amsterdam, Netherlands; 3 Department of Surgery, Leiden University Medical Centre, Leiden, Netherlands; 4 Department of Radiology, Academic Medical Centre, Amsterdam, Netherlands; 5 Department of Medical Genetics, University Medical Centre, Utrecht, Netherlands; 6 Department of Surgery, Academic Hospital, Maastricht, Netherlands; 7 Department of Surgery, Radboud University, Nijmegen, Netherlands; 8 Department of Public Health, Erasmus University Medical Centre, Rotterdam, Netherlands; 9 Department of Medical Oncology, Erasmus University Medical Centre-Daniel den Hoed Cancer Centre, Rotterdam, Netherlands; 10 Department of Genetics, University of Groningen, UMCG, Groningen, Netherlands; 11 Department of Radiology, Erasmus University Medical Centre, Rotterdam, Netherlands

**Keywords:** Breast cancer, Familial risk, Screening, MRI, Breast density, Cost-effectiveness

## Abstract

**Background:**

To reduce mortality, women with a family history of breast cancer often start mammography screening at a younger age than the general population. Breast density is high in over 50% of women younger than 50 years. With high breast density, breast cancer incidence increases, but sensitivity of mammography decreases. Therefore, mammography might not be the optimal method for breast cancer screening in young women. Adding MRI increases sensitivity, but also the risk of false-positive results. The limitation of all previous MRI screening studies is that they do not contain a comparison group; all participants received both MRI and mammography. Therefore, we cannot empirically assess in which stage tumours would have been detected by either test.

The aim of the Familial MRI Screening Study (FaMRIsc) is to compare the efficacy of MRI screening to mammography for women with a familial risk. Furthermore, we will assess the influence of breast density.

**Methods/Design:**

This Dutch multicentre, randomized controlled trial, with balanced randomisation (1:1) has a parallel grouped design. Women with a cumulative lifetime risk for breast cancer due to their family history of ≥20%, aged 30–55 years are eligible. Identified BRCA1/2 mutation carriers or women with 50% risk of carrying a mutation are excluded. Group 1 receives yearly mammography and clinical breast examination (n = 1000), and group 2 yearly MRI and clinical breast examination, and mammography biennially (n = 1000).

Primary endpoints are the number and stage of the detected breast cancers in each arm. Secondary endpoints are the number of false-positive results in both screening arms. Furthermore, sensitivity and positive predictive value of both screening strategies will be assessed. Cost-effectiveness of both strategies will be assessed. Analyses will also be performed with mammographic density as stratification factor.

**Discussion:**

Personalized breast cancer screening might optimize mortality reduction with less over diagnosis. Breast density may be a key discriminator for selecting the optimal screening strategy for women < 55 years with familial breast cancer risk; mammography or MRI. These issues are addressed in the FaMRIsc study including high risk women due to a familial predisposition.

**Trial registration:**

Netherland Trial Register NTR2789

## Background

A positive family history is one of the most important risk factors for breast cancer [1]. Women with a family history of breast cancer are not only at greater risk of developing breast cancer, but their risk also increases at a younger age than in the general population [2]. In over 75% of the families that display clear clustering of breast cancer no causative gene mutation like BRCA1 or BRCA2 can be detected [3]. Tumour stage at detection is of key influence on survival [4]. Aiming at early detection and ultimately to reduce mortality risk, women, with a positive family history for breast cancer, are often offered annual screening with mammography before age 50 [5-7]. However, screening also causes false-positive test results.

In the last decade several screening trials in high-risk women have been completed and Magnetic Resonance Imaging (MRI) had a significantly higher sensitivity for invasive breast cancer than mammography in all studies [8-12]. However, MRI was expensive and was associated with significantly more false-positive results in most studies. Furthermore, mammography had better sensitivity for the pre-invasive stage of breast cancer: ductal carcinoma in situ (DCIS) [13]. Therefore, mammography should perhaps not be omitted completely when MRI screening is offered.

Despite the higher costs of MRI and the false-positive results, screening with yearly MRI in addition to mammography is considered cost-effective for female *BRCA1* and *BRCA2* gene mutation carriers aged 30–60 years or women who have a 50% chance of carrying such a mutation [14-16]. For women with a familial risk, from families without a proven genetic predisposition, results are inconclusive [17,18]. Since previous screening studies have performed MRI and mammography simultaneously the difference in stage of the tumours when detected by mammography alone is not known. A randomized controlled trial is therefore needed.

Apart from a positive family history and age, high breast tissue density is a well documented risk factor for breast cancer. Breast density increases breast cancer incidence significantly [19,20]. At the same time, high mammographic density impairs the sensitivity of mammography [19-22], but far less the sensitivity of MRI [23]. The lower sensitivity of mammography in dense breasts is most likely caused by a masking effect, rather than by a higher tumour growth rate in denser tissue [21,24]. Breast density is high or very-high in about 50-74% of women between 40 to 49 years of age, whereas only 20-44% of women in their 60s have dense or extremely dense breast tissue [25,26]. This dual effect of breast density on cancer incidence and sensitivity of mammography results in women with the highest risk being screened with a tool with limited effectiveness: mammography.

To the best of our knowledge, no study has been published assessing the cost-effectiveness of MRI specifically in women with a familial risk for breast cancer, without a known genetic predisposition. Therefore, guidelines for breast cancer screening for women with a familial risk vary widely internationally and are weakly underpinned. The 2008 American Cancer Society and 2010 American College of Radiologists guidelines advise MRI screening for women with a familial cumulative lifetime risk (CLTR) > 20% [18], while the Dutch guidelines advise screening with mammography only [17].

Robust cost-effectiveness analyses cannot be based on the published studies, as all had a paired design (i.e. all participants received both mammography and MRI). These studies cannot examine the improvement in tumour stage at diagnosis, as one cannot know in what stage the tumour would have been diagnosed by either test alone. A randomized controlled trial is needed for a valid answer to these questions.

Furthermore, cost-effectiveness of either imaging technique may vary across categories of mammographic density. Breast density has not yet been evaluated as a parameter to identify sub-groups of women with a familial risk, for whom MRI is cost-effective. A prospective randomized trial in women with increased breast cancer risk, taking breast density into account, will give robust evidence on which screening tool, MRI or mammography, is best suited for a particular woman. These issues are addressed in the Familial MRI Screening study (FaMRIsc).

## Methods/Design

### Trial design

The FaMRIsc study is a multicentre, randomized controlled trial (RCT), with balanced randomisation (1:1), and a parallel group design conducted in the Netherlands. The study is in compliance with the Helsinki declaration and ethical approval has been granted on 8 November 2010 by the Institutional Review Board of the Erasmus University Medical Centre, Rotterdam, the Netherlands (reference-number: MEC-2010-292).

### Participants

Eligible participants are women aged 30–55 years with a cumulative lifetime risk (CLTR) of >20% because of a familial predisposition according to the modified tables of Claus [1,27] or as assessed at a Clinical Genetics Centre. *BRCA1* and/or *BRCA2* mutation carriers or women with a 50% likelihood of such a mutation are excluded, since MRI screening is already advised for these women by the American Cancer Society (ACS), the American College of Radiologists (ACR), the United Kingdom’s NICE guideline and the European guideline of the European Society of breast imaging (EUSOBI) [18,28-30]. Exclusion criteria are previous invasive cancer (potentially of influence on survival data), and a contraindication for contrast-enhanced MRI (decreased creatinin clearance, metal implants or claustrophobia).

### Study settings

Participants are recruited from outpatient breast or family cancer clinics at all eight academic medical centres in the Netherlands and the Netherlands Cancer Institute/Antoni van Leeuwenhoek Hospital ( Additional file 1). Women who are already in a screening programme because of an increased familial risk and meeting inclusion criteria are sent study information 2 weeks before a scheduled visit. Women who meet all criteria and visit the outpatient clinic for an initial screening are given information on site.

### Interventions

After informed consent is obtained participants are randomized through a computer-generated randomization sequence with stratification for centre, in one of the two groups.

Group 1 receives screening according to the 2012 Dutch guidelines [17] with yearly mammography and clinical breast examination (CBE).

**Figure 1:**
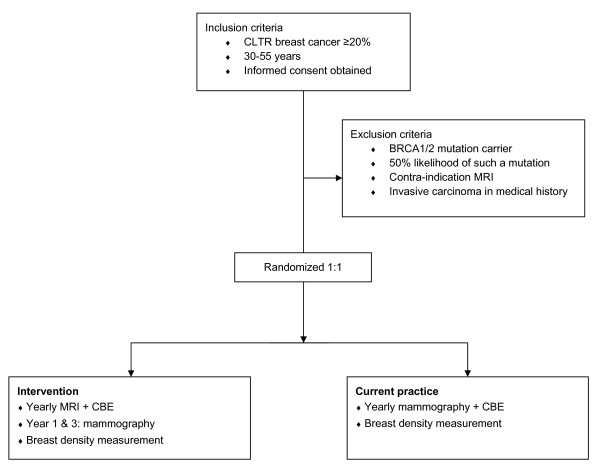
** Flow diagram Familial MRI Screening Study (FaMRIsc).** CLTR: *Cumulative Lifetime Risk,* MRI: *Magnetic Resonance Imaging*, CBE: *Clinical Breast Examination*.

Group 2 is screened with yearly MRI and CBE, and mammography biennially (Figure 1). Additional investigations are performed if deemed necessary due to findings at clinical examination, on mammography or MRI. Mammography still has a place in both arms, since DCIS is generally easier to detect with mammography [8,10,31-33], although in one study MRI was found to detect more aggressive grade III DCIS than mammography [34]. In the intervention arm however, the frequency of mammography is reduced from annually to biennially. DCIS not detected by MRI will most likely be low-grade, progress slowly, and be detected by the next mammographic examination. Leaving out mammography every other year seems safe in the MRI arm and may prevent over diagnosis of low-grade DCIS. Mammographic examination is done using full field digital mammography (FFDM). All examinations are scored in a standardized way, according to the Breast Imaging Reporting and Data System (BI-RADS) mammography classification of the American College of Radiology [30]. To determine mammographic density an automated breast density measurement is done on raw data of the first FFDM of all participants [35,36]. Dynamic breast MRI with gadolinium-containing contrast medium is performed according to standard protocol. In premenopausal women, the MRI is performed between day 5 and 20 of the menstrual cycle [37]. 

### Outcomes

Primary endpoints are the number and stage of detected breast cancers, both DCIS and invasive, in each arm. Secondary endpoints are the false-positive results in the screening arms, and the sensitivity and positive predictive value of both screening strategies. Furthermore, cost-effectiveness and breast cancer mortality reduction of both strategies will be assessed. All analyzes will also be performed stratified for mammographic density.

A positive screening test is defined as a mammographic or MRI examination with a BI-RADS score of 0, 3, 4, or 5 and/or a clinical breast examination classified as ‘suspicious’. Interval cancers are defined as tumours diagnosed after a negative screening examination but before the next scheduled screening examination. Sensitivity is calculated as the number of screen-detected tumours divided by the total of screen-detected and interval tumours. The positive predictive value of a screening strategy is calculated as the proportion of women with a positive screening test, which after pathology indeed proved to be breast cancer.

Prospective assessment of mortality reduction requires a very lengthy follow-up and a large study population, which may not be feasible. To study this issue we will start with a less costly and time-consuming approach and estimate mortality reduction through a micro-simulation model: MISCAN, a well-validated micro-simulation model, originally developed to estimate the cost-effectiveness of the population-based screening program in the Netherlands [38-40]. In the model, the natural history of breast cancer is modelled as a progression through 5 pre-clinical and invasive disease stages. At each pre-clinical stage, a tumour may either be clinically diagnosed or grow into the next pre-clinical stage. Screening may detect the tumour in a preclinical stage. Transition probabilities, stage durations and survival after clinical diagnosis or screen detection are based on data from the Dutch nation-wide screening program [41,42]. The improvement of prognosis after detection by screening is based on the long-term effects of Swedish trials [43-45]. A detailed description of the model has been published previously [39].

We will develop a family history risk model by using the number of women enrolled in the study, the age distribution at entry of the study, the duration of follow-up and the screening protocol, attendance and sensitivity of different screenings methods as inputs. The model will be calibrated using the number of screens, the number of screen detected cancers and interval cancers, the stage distribution and the age at diagnosis. Likelihood ratio tests will be used to compare the goodness of fit. Using the calibrated model, predictions of the number of screens, number of screen detected and interval cancers, the stage distribution, the mortality reduction and the life years gained will be made for the different screening arms in the study.

A cohort of 5 million women will be simulated. All costs and effects will be predicted for a life-time follow-up. The costs will be presented in European currency (€). Cost-effectiveness ratios will be expressed as cost per life year gained (LYG). Costs and effects will be discounted at an annual rate of 3.5%.

### Sample size/power calculation

Our primary aim is to detect a difference in tumour stage between the intervention and the current practice group. In the Dutch MRI Screening Study (MRISC) study, conducted from 1999 to 2007, over 1500 women with familial risk were included in the 6 participating centres [11]. The incidence rate in this risk group was 7/1000 women years screened. Since the FaMRIsc study will have three more participating centres we intend to include 2000 women. We expect to detect about 50 breast cancers (both DCIS and invasive) in 4 years. With this number we are able to detect a difference in tumour size of 8 mm (SD tumour size: 9 mm) as statistically significant (two sided alpha =0.05) with a power of 80%. Eight mm is considered to be a clinically relevant difference.

### Stopping guidelines

The accrual will be evaluated after two years. If adequate inclusion numbers cannot be achieved, appropriate measures will be taken in the remaining two years, consisting of expansion of the number of participating centres or longer continuation of the study.

### Statistical methods

Primary outcome will be incidence and the difference in mean tumour size at diagnosis between the two arms. If normally distributed, this will be tested by means of the independent samples (unpaired) *t*-test. If not normally distributed, medians will be estimated and differences between distributions will be tested with the non-parametric Mann–Whitney *U* test.

Breast cancer incidence rates will be calculated as the total number of breast cancers detected per 1000 woman-years at risk. This will be calculated both including and excluding DCIS. Differences between these proportions will be compared using a chi-square test or Fisher’s exact test as appropriate.

All tests will also be performed stratified by mammographic density to examine the influence of density on the efficacy of MRI screening versus usual care. The influence of breast density on detection rates, tumour stage and false positive results in both arms will be analysed by means of analysis of variance (ANOVA).

## Discussion

Twenty-five percent of all breast cancers occur before age 50 and especially familial breast cancer is seen at younger ages [2]. A randomized controlled trial can provide the best evidence for any breast cancer mortality reduction attributable to digital mammography or MRI screening in this population.

Studies that offered MRI and mammography screening simultaneously have several shortcomings due to their paired design. Sensitivity of neither test without the other can be assessed. Nor the stage in which either test separately would have detected tumours.

With the results of our study we will be able to estimate the mortality reduction for screening women with familial risk with either digital mammography or additional MRI.

Furthermore, we will be able to assess whether mortality reduction by earlier detection differs with increasing breast density between screening with digital mammography or with additional MRI.

Breast density may be a key discriminator for choosing the optimal screening strategy below age 50 years for women with familial risk. If we can assess this, personalized cancer screening can be offered, based on a woman’s age, risk and breast density. This may optimize mortality reduction, whilst possibly decreasing over diagnosis. Compliance to screening will be best if there is convincing evidence that the most effective tool with the lowest side-effects is offered.

## Abbreviations

ANOVA: Analysis of variance; ACS: American Cancer Society; ACR: American College of Radiologists; BI-RADS: The Breast Imaging Reporting and Data System; CBE: Clinical Breast examination; CLTR: Cumulative Lifetime Risk; DCIS: Ductal Carcinoma In Situ; EUSOBI: European Society Of Breast Imaging; FaMRIsc: Familial MRI Screening Study; FFDM: Full Field Digital Mammography; LYG: Life Year Gained; MISCAN: Microsimulation Screening Analysis; MRI: Magnetic Resonance Imaging; MRISC: MRI Screening Study; RCT: Randomized Controlled Trial.

## Competing interests

The authors declare that they have no competing interests.

## Authors’ contributions

MMAT-L is the scientific coordinator of this research and designed the study. MMAT-L, IMO, HJK and SS applied for funding. MMAT-L and SS have drafted the manuscript with critical input from all other authors who have read, and approved the final manuscript. All authors are involved in data acquisition.

## Pre-publication history

The pre-publication history for this paper can be accessed here:

http://www.biomedcentral.com/1471-2407/12/440/prepub

## Supplementary Material

Additional file 1 ** Academic Medical Centres participating in the FaMRIsc in the Netherlands.** (DOC 31 kb) Click here for file
