# Supplementary material for: Breast density as indicator for the use of mammography or MRI to screen women with familial risk for breast cancer (FaMRIsc): a multicentre randomized controlled trial
Source: BMC Cancer. 2012 Oct 2;12:440. doi: 10.1186/1471-2407-12-440 (PMC3488502; doi:10.1186/1471-2407-12-440)
Supplement: Additional file 1 — Academic Medical Centres participating in the FaMRIsc in the Netherlands. (DOC 31 kb) [file 1471-2407-12-440-S1.doc]

**Additional file 1**

**Academic Medical Centres participating in the FaMRIsc in the Netherlands**

| **Academic Centre** |
| --- |
| Erasmus University Medical Centre, Rotterdam |
| The Netherlands Cancer Institute, Antoni van Leeuwenhoek Hospital, Amsterdam |
| Leiden University Medical Centre, Leiden |
| Academic Medical Centre, Amsterdam |
| University Medical Centre, Utrecht |
| Academic Medical Hospital, Maastricht |
| Radboud University, Nijmegen |
| University Medical Centre, Groningen |
| VU University Medial Centre, Amsterdam |
